# The complete genome sequence of *Sedimentibacter* sp. strain MB35-C1, isolated from the sewage sludge

**DOI:** 10.1128/mra.00064-24

**Published:** 2024-05-29

**Authors:** Rui Zhang, Chih-Hung Wu, Chao-Jen Shih, Yen-Chi Wu, Shu-Jung Lai, Yi-Ting You, Sheng-Chung Chen

**Affiliations:** 1 College of Environment and Resources, College of Carbon Neutral and Modern Industry, Fujian Normal University, Fuzhou, Fujian, China; 2 School of Resources and Chemical Engineering, Sanming University, Sanming City, Fujian, China; 3 Fujian Provincial Key Laboratory of Resources and Environmental Monitoring and Sustainable Management and Utilization, Sanming University, Sanming, Fujian, China; 4 Bioresource Collection and Research Center, Food Industry Research and Development Institute, Hsinchu, Taiwan, Republic of China; 5 Graduate Institute of Biomedical Sciences, China Medical University, Taichung City, Taiwan, Republic of China; 6 Research Center for Cancer Biology, China Medical University, Taichung City, Taiwan, Republic of China; 7 Department of Life Sciences, National Chung Hsing University, Taichung City, Taiwan, Republic of China; SUNY College of Environmental Science and Forestry, Syracuse, New York, USA

**Keywords:** *Sedimentibacter*, sewage sludge

## Abstract

Here, we report the complete genome sequence of *Sedimentibacter* sp. strain MB35-C1, which was isolated from sewage sludge at the Wastewater Treatment Plant of Sanming Steel Co. Ltd. in Fujian, China. The resulting genome of strain MB35-C1 is a single contig of 3,621,605 bp.

## ANNOUNCEMENT

Strain MB35-C1 was isolated from sewage sludge of the Wastewater Treatment Plant of Sanming Steel Co. Ltd, Fujian, China. The sludge was collected from the dehydrated sludge on 25 June 2021 and then inoculated into the anaerobic modified DSM 924 medium (per liter: 5 g NaCl; 1 g MgCl_2_·6H_2_O; 0.5 g KCl; 0.1 g CaCl_2_·2H_2_O; 0.4 g K_2_HPO_4_; 1 g NH_4_Cl; 10 mL trace element solution (1); 2 g yeast extract; 2 g trypticase peptone; 0.5 mL 0.1% resazurin solution; 4 g NaHCO_3_; 10 mL vitamins solution; 0.25 g L-cysteine-HCl·H_2_O; 0.25 g Na_2_S·9H_2_O, prepared under gas phase of N_2_:CO_2_ = 80:20) and incubated at room temperature (~25°C) for 2 weeks. Strain MB35-C1 was further purified and identified using the method combined with serial dilution, rolling-tube technique (2), and 16S rRNA gene clone sequencing with 8F (5′-AGAGTTTGATCCTGGCTCAG-3′) and 1492RU (5′-TTTTAATTAAGGTTACCTTGTTACGACTT-3′) primers (3). The purity of bacteria was verified by observing morphology and conducting 16S rRNA gene and genome sequencing. Based on the analysis using the EZBioCloud 16S rRNA gene database (4), strain MB35-C1 exhibited the highest similarities to three characterized and validly strains: *Sedimentibacter hydroxybenzocus* JW/Z-1^T^ (95.90%) (5, 6), *Sedimentibacter saalensis* DSM 13558^T^ (95.87%) (6), and *Sedimentibacter acidaminivorans* MO-SEDI^T^ (95.33%) (7). These results indicate that strain MB35-C1 belongs to the genus *Sedimentibacter*. The genome of strain MB35-C1 was sequenced for species delineation and comparative genomic analysis.

Strain MB35-C1 was cultured in modified DSM 924 medium at 35°C for 2 days. The genomic DNA was extracted from a cell pellet collected from 1 L culture using the NucleoBond HMW DNA kit (Macherey-Nagel, Germany) following the manufacturer’s instructions. The genome was sequenced at the Sangon Biotech (Shanghai) Co., Ltd. using the DNBSEQ-T7 platform (MGI Tech Co., Ltd.) and the MinION sequencer (Oxford Nanopore Technology).

For the DNBSEQ-T7 platform, sheared genomic DNA fragments (approximately 300 bp), sheared by sonication using Covaris, were utilized to prepare a 150 bp paired-end DNA library. The library preparation was performed using the Hieff NGS MaxUp II DNA Library Prep Kit for Illumina [Yeasen Biotechnology (Shanghai) Co., Ltd.]. The constructed library was sequenced using MGISEQ-2000RS High-throughput Sequencing Set (PE150 format), and 9,208,398 reads were generated and trimmed using Trimmomatic v0.36 (8). For the MinION sequencer, the original extracted genomic DNA underwent a series of processing steps including end-repair, 3’ adenylation, and ligation to adaptors pre-loaded with motor proteins. The resulting product was then purified using Agencourt AMPure XP Beads (Beckman, A63881). Subsequently, fragments larger than 1 Kb were subjected to single-molecule nanopore DNA sequencing using the Multiplex Ligation Sequencing Kit (SQK-MLK111.96-XL) on a MinION Flow Cell (R9.4.1). Basecalling was performed using Guppy version 6.5.6 with the default model, yielding a total of 229,034 reads and *N*_50_ of 6,328 bp. Trimming of the reads was carried out using Porechop v0.2.4 (9), and filtering was executed with NanoFilt v2.8.0 (10). DNBSEQ-T7 and MinION reads were hybrid *de novo* assembled using Canu v2.2 (11), producing a circularized contig of 3,621,605 bp with 35.43% GC content. Gene predictions and annotations were performed using NCBI Prokaryotic Genome Annotation Pipeline v6.5 (12). Default parameters were used for all bioinformatics analyses. Table 1 provides general features of this strain and genome.

**TABLE 1:** General features of *Sedimentibacter* sp. strain MB35-C1 based on MIGS (Minimum Information for Genome Sequence) recommendation and general information on its genome

Item	Description
MIGS data	
NCBI BioProject	PRJNA1006667
NCBI BioSample	SAMN37044515
GenBank accession number	CP133188.1
Geographic location	Sewage sludge of Wastewater Treatment Plant of Sanming Steel Co. Ltd., Sanming City, Fujian, China
Latitude and longitude	26.2649 N, 117.6245 E
Collection date	25 June 2021
Sequencing platforms	DNBSEQ-T7 and Oxford Nanopore MinION
Assembly method	Canu v. 2.2
Coverage	889.0×
Finishing strategy	Complete
General features of strain	
Classification	Domain: *Bacteria* Phylum: *Bacillota* Class: *Clostridia* Order: *Tissierellales* Family: *“Sedimentibacteraceae”* Genus: *Sedimentibacter*
Gram stain	Positive
Cell shape	Rod-shaped
Motility	Non-motile
Relationship to oxygen	Anaerobic
Optimal growth temperature	35°C
Optimal growth pH range	7.5
Optimal growth NaCl concentration	0.5%
Genomic features	
Size (bp)	3,621,605 bp
GC content (%)	35.43
Coding DNA Sequences (CDSs)	3,414
Number of tRNAs	84
Number of rRNAs	7, 6, and 6 (5S, 16S, and 23S)

## Data Availability

The genome sequence of strain MB35-C1 has been deposited in GenBank under accession number CP133188. The version of the genome described in this paper is first version. The BioProject and BioSample accession numbers are PRJNA1006667 and SAMN37044515. DNBSEQ-T7 and MinION raw reads were deposited in the Sequence Read Archive (SRA) under accession numbers SRR27218472 and SRR27218471, respectively.
